# Risk factors and characteristics of blood stream infections in patients with newly diagnosed multiple myeloma

**DOI:** 10.1186/s12879-016-2155-1

**Published:** 2017-01-06

**Authors:** Chun-Teng Huang, Chia-Jen Liu, Po-Shen Ko, Han-Tsung Liu, Yuan-Bin Yu, Liang-Tsai Hsiao, Jyh-Pyng Gau, Cheng-Hwai Tzeng, Tzeon-Jye Chiou, Jin-Hwang Liu, Muh-Hwa Yang, Ling-Ju Huang, Chun-Yu Liu

**Affiliations:** 1 Division of Hematology & Oncology, Department of Medicine, Taipei Veterans General Hospital, Taipei, Taiwan; 2 National Yang-Ming University School of Medicine, Taipei, Taiwan; 3 Division of Transfusion Medicine, Department of Medicine, Taipei Veterans General Hospital, Taipei, Taiwan; 4 Division of Hematology & Oncology, Department of Medicine, Yang-Ming Branch of Taipei City Hospital, Taipei, Taiwan; 5 Division of General Medicine, Department of Medicine, Taipei Veterans General Hospital, No.201, Sec. 2, Shipai Rd., Beitou District, Taipei City, 11217 Taiwan; 6 Division of Medical Oncology, Department of Oncology, Taipei Veterans General Hospital, Taipei, Taiwan

**Keywords:** Bloodstream infection, Multiple myeloma, Risk factor

## Abstract

**Background:**

Patients with multiple myeloma are generally immune-compromised either due to pronounced depression in primary antibody responses or because of anti-myeloma therapy. Infection is a major risk factor for early deaths among these patients. The impact of blood stream infections (BSI) on newly diagnosed myeloma patients has been less studied. We aimed to study the incidence and risk factors of BSI within 3 months after diagnosis of multiple myeloma in a tertiary referral center.

**Methods:**

Between November 2002 and December 2008, consecutive patients with multiple myeloma in Taipei Veterans General Hospital were retrospectively enrolled. Characteristics of patients with or without BSI were collected. Possible factors associated with development of BSI were analyzed by Cox regression.

**Results:**

There were a total of 222 patients. The incidence of BSI within 3 months after diagnosis is 11.7%. The patients with BSI had poorer survival outcomes than those without (mortality rate: 50% vs. 20.9%, *p* < 0.001). Moreover, advanced International Staging System stage (stage III vs. I/II: odds ratio [OR] 2.69, *p* = 0.049) and poor Eastern Cooperative Oncology Group (ECOG) performance status (ECOG > 2 vs. ≤ 2: OR 3.58, *p* = 0.005) were the independent risk factors of BSI, whereas immunoglobulin deficiency and low absolute lymphocyte count were not associated with risk of BSI development.

**Conclusions:**

Our study highlights the characteristic of myeloma patients with BSI and the importance of disease and host factors on risk of BSI. Myeloma patients with risks of BSI should be properly managed to reduce early mortality.

**Electronic supplementary material:**

The online version of this article (doi:10.1186/s12879-016-2155-1) contains supplementary material, which is available to authorized users.

## Background

Early death in multiple myeloma (MM) is usually attributed to combined effects such as old age, active disease, comorbidities, and anti-myeloma therapy. Earlier series had showed 10 to 25% of patients dying within 6 months of diagnosis [1–3]. Auguston et al. reported a incidence of 10% (299/3107) patients die within 60 days of diagnosis of MM, and the major cause of death are infection and renal failure [4].

The increased susceptibility to bacterial infection in MM patients is complicated and multifactorial. Though impaired antibody production is very likely to be the main cause of infection at disease onset [5], other factors such as poor response following immunization, renal function impairment, defective opsonization, decreased granulocyte adhesiveness, impaired leukocyte migration, chemotherapy associated granulocytopenia, and high-dose dexamethasone all play their roles [6]. Infection is common in patients with newly diagnosed multiple myeloma and can be the initial presentation and the leading cause of death. In an earlier study, pulmonary and urinary tract infection occur more frequently [7], and infection not only occurs as the presenting feature in about 15% of patients with MM, but also is the leading cause of mortality. In another recent study, among the multiple myeloma patients with bacterial infection as the initial presentation, musculoskeletal infections and pneumonia are the leading cause of infection [8].

With the progress of anti-myeloma therapy and best supportive care, major changes have occurred in the spectrum of causative organisms. The relationship between bloodstream infection (BSI) with associated pathogen and early mortality in newly diagnosed MM, however, is too few to be mentioned in literature. The main aim of this study is to determine the impact of BSI on the outcome of newly diagnosed MM patients and the risk factors of BSI. We believe that the knowledge of this analysis will raise awareness of early mortality seen in myeloma and may help with developing strategies for its prevention and treatment.

## Methods

### Data collection

This study was approved by the Institutional Review Board of Taipei Veterans General Hospital. Between November 2002 and December 2008, consecutive patients diagnosed with multiple myeloma in Taipei Veterans General Hospital were eligible for retrospective screening. A total of 252 patients were enrolled. There were 30 patients that were excluded due to being previously treated at other hospitals or for not being consistent with the criteria for symptomatic myeloma as defined and published by the International Myeloma Working Group [9]. Patients aged < 65 years old, or candidates for autologous stem cell transplantation (ASCT), received either vincristine/doxorubicin/dexamethasone (VAD) based regimen, thalidomide based regimens, or bortezomib based regimen as induction chemotherapy. In contrast, patients aged ≧ 65 years old, or not a candidate for ASCT received either melphalan/prednisolone (MP), thalidomide based regimens or bortezomib based regimen as palliative chemotherapy. Patients’ demographic data and clinical characteristics were obtained from clinical chart review. Complete blood count, serum biochemistries, myeloma stage, performance status, blood culture results were collected.

### Definitions

BSI was defined according to the Centers for Disease Control and Prevention/National Healthcare Safety Network (CDC/NHSN) guidelines as positive blood culture related to a febrile episode [10]. In order to explore the impact of BSIs on early mortality of patients with multiple myeloma, only blood stream infections within 90 days from the diagnosis of multiple myeloma were documented. Two bottle cultures were obtained routinely in each febrile episode. Early mortality was calculated from date of diagnosis of multiple myeloma to date of death or date last seen, as appropriate, and was defined as death within 100 days.

Definite catheter-related blood stream infection (CRI) was defined as bacteremia or fungemia in a patient with the central vascular catheter (CVC) with at least one positive blood culture obtained from a peripheral vein, clinical manifestations of infection (e.g. fever, chills, hypotension), and no apparent source of the BSI aside from the catheter. Nosocomial infection was defined as infection that becomes evident 48 h or more after admission. Severe immunoglobulin deficiency was defined as both of the non-myeloma immunoglobulin levels being less than one-fourth of the lower normal limit. Central vascular access device were grouped into non-tunneled catheter and tunneled catheter. Non-tunneled catheter included internal jugular vein central venous catheter and femoral vein catheter. Tunneled catheter included implanted port and Perm cath.

### Statistical analysis

Patients were separated into BSI (developed within 3 months of diagnosis of myeloma) and non-BSI groups. Data and survival analyses were compared between two groups. Early mortality (within 100 days of diagnosis of multiple myeloma) was the end point. PASW statistics software (version 18.0, SPSS, Chicago, Illinois) was used for all statistical analysis. The correlations of variables between the BSI and non-BSI groups were analyzed by a Chi-square test. Overall survival (OS) was measured from the date of diagnosis of multiple myeloma to death of any cause. Survival was estimated using the Kaplan-Meier method and the log-rank test was used for comparison of survival curves. The Cox proportional hazards model was applied for univariate and multivariate analyses to determine the prognostic influence of variables on development of BSI. Variables with *p* < 0.05 in univariate analysis were considered in the multivariate analysis. A *P* value < 0.05 in two-tailed tests was considered statistically.

## Results

### General characteristics

There were 26 BSI patients (11.7%) in this study. Baseline characteristics of the 26 BSI and 196 non-BSI patients were listed in Table 1. Between two groups, there was no statistical difference in gender, age, myeloma types, status of immunoglobulin deficiency, absolute lymphocyte count (ALC), and induction chemotherapy. In the BSI group, patients tended to have a more advanced stage (ISS stage III, 77% : 48%, *p* = 0.019), poor ECOG performance status (>2, 65% : 30%, *p* < 0.001), anemia (hemoglobin <10 g/dL, 81% : 55%, *p* = 0.015), hypercalcemia (serum calcium > 12.0 mg/dL, 19%: 7%, *p* = 0.038), and renal dysfunction (serum creatinine ≥ 2.0 mg/dL, 58% : 28%, *p* = 0.002).

**Table 1:** Baseline characteristics of 222 myeloma patients

	No. of patients (%)				*P* value^*^
	Non-BSI (*n* = 196)		BSI (*n* = 26)		
Gender					
Male	141	(72)	17	(65)	0.492
Female	55	(28)	9	(35)	
Age ≥ 65 y/o	127	(65)	19	(73)	0.402
Myeloma subgroup					
IgG	95	(48)	13	(50)	0.741
IgA	63	(32)	9	(35)	
Light chain disease	29	(15)	4	(15)	
Other types^a^	9	(5)	0	(0)	
Immunoglobulin status					
Severe Ig deficiency^b^	177	(90)	23	(88)	0.767
Others	19	(10)	3	(12)	
ALC count ≥ 1000 (/ul)	138	(70)	17	(65)	0.612
ISS Stage					
I	37	(19)	1	(4)	
II	64	(33)	5	(19)	
III	95	(48)	20	(77)	0.019
ECOG PS >2	59	(30)	17	(65)	<.001
Hb < 10 (g/dL)	108	(55)	21	(81)	0.015
Ca > 12.0 (mg/dL)	14	(7)	5	(19) (58)	0.038
Cr ≥ 2.0 (mg/dL)	55	(28)	15		0.002
Induction chemotherapy					
VAD-based	57	(29)	13	(50)	
MP-based	83	(42)	7	(27)	0.064
Others^c^	30	(15)	1	(4)	
No	26	(13)	5	(19)	

*Abbreviations*: *BSI* Blood stream infection, *IgG* Immunoglobulin G, *IgA* Immunoglobulin A, *Ig* Immunoglobulin, *ALC* Absolute lymphocyte count, *ISS* International Staging System, E*COG PS* Eastern Cooperative Oncology Group Performance Status, *Hb* Hemoglobin, *Ca* Calcium, *Cr* Creatinine, *VAD* Vincristine/doxorubicin/dexamethasone, *MP* Melphalan/prednisolone

^a^Other types include solitary plasmacytomas and plasma cell leukemia

^b^Severe Ig deficiency is defined as both of the non-myeloma immunoglobulin levels less than one-fourth of lower normal limit

^c^Including newer regimens such as thalidomide/dexamethasone (TD), bortezomib/cyclophosphamide/dexamethasone (BCD), bortezomib/dexamethasone (BD), cyclophosphamide/thalidomide/dexamethasone (CTD), and bortezomib/cyclophosphamide/dexamethasone (BTD)

^*^Statistics analysis is used Chi-square or Fisher exact test

### Outcome analysis

**Fig. 1:**
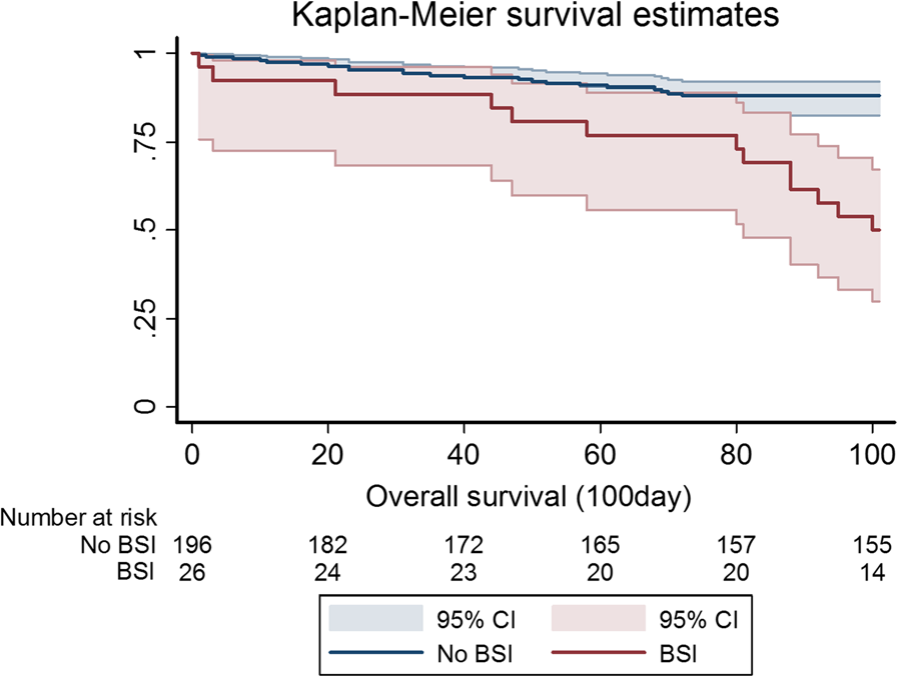
Kaplan-Meier survival curves according to blood stream infection (BSI) or not. Myeloma patients who had BSI had poorer post-diagnosis 100-day survival outcome than non-BSI patients (100-day mortality rate: 50% vs. 20.9%, *p* < 0.001). CI Confidence interval

**Fig. 2:**
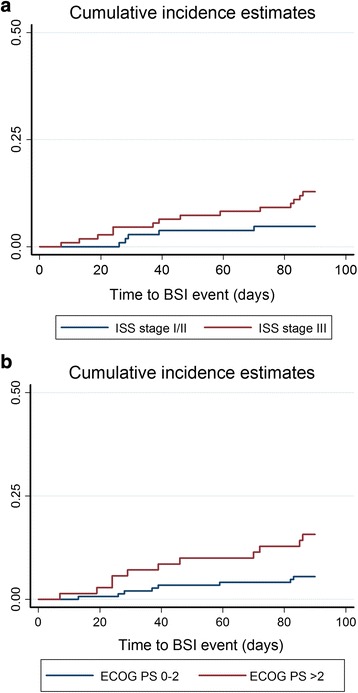
Cumulative incidence curves according to myeloma ISS stage (**a**) or ECOG performance status (**b**). The cumulative incidence of BSI is significantly higher in patients with either more advanced stage (*p* = 0.006) (Fig. 2a) or poor ECOG performance status (*p* < 0.001) (Fig. 2b). Note early deaths (within 90 days) were the competing events in the analysis

Overall, 24.3% (54/222) of newly diagnosed MM patients died within 100 days after diagnosis. Thirteen of 26 BSI patients died within 100 days. Patients who had BSI within 3 months had poorer survival outcomes than those without (mortality rate: 50% vs. 20.9%, *p* < 0.001) (Fig. 1). Cumulative incidence of BSI is significantly higher in patient with either more advance stage (*p* = 0.006) (Fig. 2a) and poor ECOG performance status (*p* < 0.001) (Fig. 2b).

### Risk factors of BSI

Univariate analysis displayed more advanced stage, poor performance status, more severe anemia, and worse renal function as possible risk factors associated with BSI. However, absolute lymphocyte count (ALC), < 1000/cumm vs. ≥ 1000/cumm, did not showed significant difference. Moreover, multivariate analysis showed only advanced ISS stage (ISS stage III vs. stage I/II: odds ratio [OR] 2.69, *p* = 0.049) and poor ECOG performance status (ECOG PS score > 2 vs. ≤ 2: OR 3.58, *p* = 0.005) were the independent risk factors of BSI (Table 2).

**Table 2:** Univariate and multivariate analysis for blood stream infection

	Univariate			Multivariate		
	Odds ratio	95% CI	*P* value*	Odds ratio	95% CI	*P* value*
Gender (female vs. male)	1.36	0.571–3.266	0.49	-	-	-
Age (≥ 65 vs. < 65)	1.48	0.591–3.681	0.40	-	-	-
Myeloma subgroup						
IgG vs. Non-IgG	1.06	0.469–2.410	0.88	-	-	-
IgA vs. Non-IgA	1.12	0.472–2.646	0.80			
Immunoglobulin status	1.18	0.324–4.303	0.80	-	-	-
Severe Ig deficiency^a^ vs. Others						
ISS stage						
III vs. I/II	3.54	1.365–9.203	<0.01*	2.69	1.003–7.190	0.049
ECOG PS						
Score > 2 vs. 0–2	4.39	1.849–10.403	<0.01*	3.58	1.478–8.690	0.005
Hb (< 10 g/dL vs. ≥ 10 g/dL)	3.422	1.240–9.444	0.02*	-	-	-
Ca (> 12 mg/dL vs. < 12 mg/dL)	3.095	1.013–9.454	0.04*	-	-	-
Cr (≥ 2.0 mg/dL vs. < 2.0 mg/dL)	3.091	1.341–7.126	0.01*	-	-	-
ALC (< 1000/cumm vs. ≥ 1000/cumm)	1.251	0.527–2.968	0.61			

*Abbreviations*: *IgG* Immunoglobulin G, *IgA* Immunoglobulin A, *Ig* Immunoglobulin, *ISS* International Staging System, *ECOG PS* Eastern Cooperative Oncology Group Performance Status, *Hb* Hemoglobin, *Ca* Calcium; *Cr* Creatinine, *ALC* Absolute lymphocyte count

^a^Severe Ig deficiency: both of the non-myeloma immunoglobulin levels less than one-fourth of lower normal limit

*Significant *p* < 0.05 in univariate models were entered in the Cox regression multivariate model using conditional backward analysis

### Characteristics of BSI patients

There were 26 patients that developed BSI within 3 months of diagnosis, among whom 12 patients (46%) received CVC placement during their diagnosis for various indications such as chemotherapy, fluid resuscitation, dialysis, and access for transfusion or drug administration. Two-thirds (8 of 12) of the CVC was non-tunneled. Definite catheter-related infection (CRI) was documented in three cases. Patients with CVC placement had a numerically higher early mortality (8/12, 66.6%) compared to those without (5/14, 35.7%). Nosocomial infection accounted for seventy percent (18 of 26) of the BSI events. Early death occurred in 61% (11 of 18) of patients with nosocomial BSI and in 25% (2 of 8) of patients with community-acquired BSI. Sixteen patients (61.5%) had BSI event after induction chemotherapy.

### BSI pathogens and antibiotics susceptibility

Total 27 causative pathogens consisting of 15 (55.6%) Gram-negative bacilli (GNB), 10 (37.0%) Gram-positive microorganisms, and 2 (7.4%) fungi were isolated from 26 BSI patients, among which one patient had mixed causative pathogens (Table 3). Coagulase negative staphylococcus (CNS) (*n* = 6) was the most common pathogens followed by Escherichia coli (E. coli) (*n* = 5). No significant difference in either early mortality or OS existed among Gram-positive BSI, Gram-negative BSI or fungemia (data not shown). For nosocomial BSIs (*n* = 18, 69%), the frequency of GNB was higher, up to 72.2% (13/18), and common isolates was E. coli (*n* = 4) followed by Klebsiella pneumoniae (K. pneumoniae) (*n* = 3). For community-acquired BSIs (*n* = 8, 31%), Gram-positive microorganisms (66.7%) were predominant, and CNS (*n* = 4) was the most common pathogen. The antibiotic susceptibility of the causative organisms was shown in Additional file 1.

**Table 3:** Characteristics of blood streams infections in newly diagnosed myeloma patients

Serial number	BSI after ICT	ICT regimen	Catheter type	Definite CRI^b^	Pathogen	Empirical antibiotics	Early death (< 100day)
Nosocomial BSI^a^							
1 (90)	-	-	-	-	*Coagulase-negative Staphylococcus species*	Ceftazidime/isepamicin	Y
2 (215)	Y	Thalidomide/dexamethasone	-	-	*Coagulase-negative Staphylococcus species*	Ceftazidime	-
3 (79)	Y	VAD	Implanted port	Y	*Coagulase-negative Staphylococcus species* *Klebsiella pneumoniae*	Teicoplanin/isepamicin	Y
4 (27)	-	MP	-	-	*Klebsiella pneumoniae*	Cefepime	-
5 (62)	Y	MPT	Non-tunneled CVC	Y	*Klebsiella pneumoniae*	Ciprofloxacin - > Imipenem/teicoplanin	Y
6 (78)	-	-	-	-	*Escherichia coli*		Y
7 (198)	Y	VAD	Non-tunneled CVC	-	*Escherichia coli*	Cefuroxime/clindamycin	Y
8 (168)	Y	VAD/thalidomide	Non-tunneled PICC	-	*Escherichia coli*	Imipenem/vancomycin/amikin	Y
9 (175)	Y	VAD	-	-	*Escherichia coli*	Cefuroxime/metronidazole	-
10 (150)	Y	VAD	-	-	*Enterobacter gergoviae*	Oxacillin	-
11 (14)	Y	VAD	Implanted port	-	*Enterobacter cloacae*		-
12 (94)	-	VAD	Non-tunneled CVC	-	*Pseudomonas Aeruginosa*	Cefepime	Y
13 (115)	Y	VAD	Implanted port	-	*Proteus mirabilis*	Levofloxacin	-
14 (69)	Y	VAD	-	-	*Salmonella Enteritidis group D*	Ceftazidime	Y
15 (114)	Y	VAD	Non-tunneled CVC	-	*Staphylococcus aureus*		-
16 (71)	-	-	Non-tunneled CVC	Y	*Serratia marcescens*		Y
17 (61)	Y	MP	Non-tunneled CVC	-	*Yeast like-Trichosporon asahii*	Cefepime/azithromycin	Y
18 (18)	Y	VAD	-	-	*Yeast like-Candida albicans*	Ceftazidime/teicoplanin	Y
Non-nosocomial BSI							
1 (221)	-	-	-	-	*Coagulase-negative Staphylococcus species*	Flomoxef	-
2 (41)	Y	VAD	-	-	*Coagulase-negative Staphylococcus species*	Piperacillin/tazobactam	-
3 (211)	Y	MP	-	-	*Coagulase-negative Staphylococcus species*		-
4 (205)	-	-	-	-	*Streptococcus*	Meropenem/vancomycin/metronidazole	Y
5 (137)	-	-	-	-	*Streptococcus pneumoniae*		-
6 (6)	-	MP	-	-	*Escherichia coli*	Cephalexin/isepamicin	-
7 (106)	-	VAD	Non-tunneled CVC	-	*Pseudomonas aeruginosa*	Cefoperazone	-
8 (46)	Y	MP	Tunneled CVC (Permcath)	-	*Lactobacillus species*	Moxifloxacin	Y

*Abbreviations*: *BSI* Blood stream infection, *ICT* Induction chemotherapy, *CRI* Catheter related infection, *VAD V*incristine/doxorubicin/dexamethasone, *MP* Melphalan/prednisolone, *MPT* Melphalan/prednisolone/thalidomide, *CVC* Central venous catheter, *PICC* Peripherally inserted central catheter

^a^Nosocomial infection is defined as infection become evident 48 h or more after admission

^b^Definite CRI (catheter related infection) is defined as bacteremia/fungemia in a patient with an intravascular catheter with at least one positive blood culture obtained from a peripheral vein, clinical manifestations of infection (i.e., fever, chills, and/or hypotension), and no apparent source for the bloodstream infection except the catheter

## Discussion

Despite advances in anti-myeloma therapy and supportive care, including the empirical administration of broad-spectrum antibiotics, effective management of hypercalcemia with adequate hydration and intravenous bisphosphonates, and emergent dialysis for acute renal failure, pain control, and blood transfusion, up to 24.3% (54/222) of newly diagnosed MM patients still die within 100 days of their diagnosis in this study. Such deaths are thought to occur before the maximal beneficial effect of chemotherapy in reducing tumor load and are complicated with other risk factors, such as comorbidities, infectious complications, and adverse effects caused by treatment itself. The early death rate in current study is considerably higher than previous reported; one important reason is that a large portion of patients in advanced stage and high ECOG performance status. Compare with the largest case study published by Greipp et al. in 2005 [11], we have more patients in ISS stage III (51.8% vs. 39%), and they are older (median age: 72.5 years vs. 60 years). The incidence of patients with age > 80 years is up to 18%. Furthermore, poor ECOG performance status > 2 and hemoglobin < 10 account for 34.2 and 58.1% in all patients respectively. Taipei Veterans General Hospital is a tertiary academic medical center which accepts final transfer of high risk patients and a large portion of our patient resources are veterans. These parameters can explain why early mortality rate is higher than expected.

BSI is an important cause of morbidity and mortality in populations with hematological malignancies, and may contribute to delayed administration of chemotherapy, increased length of hospitalization, and increased healthcare expenditure [11, 12]. However, to our knowledge, the relationship between BSI and early mortality in MM was seldom reported in literature. In this study, 26 patients (11.7%) of 222 patients developed BSI within 3 months of newly diagnosed MM and resulted in early mortality in 13 patients among whom. The mortality within 100 days after diagnosis of MM patients with and without bacteremia is 50 and 20.9%. Significantly, the survival analysis showed a higher rate of early mortality in patients with BSIs as compared to those without BSIs. (*p* < 0.001, Fig. 1). In multivariate analysis, only advanced ISS stage and poor ECOG performance status were the independent risk factors of BSI in newly diagnosed myeloma patients. These two features reflected the disease severity and poor medical condition, and partially explained the higher susceptibility to BSI.

The absolute lymphocyte count (ALC) has been widely studied in various malignancies as a marker of host immunity. Though its role on EM and BSI in newly diagnosed MM patients remains undetermined, the significance on survival and infection risk has been associated with multiple myeloma and other hematological malignancies during different clinical stages of disease [13–16]. In the UKMRC trials [4], there was a significant correlation between EM and lymphopenia (< 1000/cumm). However, ALC did not show any significantly difference in either the risk of BSI or EM in this study.

Multiple myeloma is characterized with an increased production of ineffective immunoglobulins with suppression of non-involved immunoglobulins. Moreover, MM cells can subvert the immune system through a wealth of potential mechanisms such as immunologically hostile microenvironment and defective cellular immunity [17]. We found that neither the type of monoclonal gammopathy nor severe non-myelomatous immunoglobulin deficiency was associated with risk of BSI. The complexity of immune dysfunction in MM may partially explain this insignificance.

Wang et al. reported that GNB accounted for 78.2% of bacteremia in 266 patients with hematologic malignancies, followed by Gram-positive cocci (GPC), 20.8%, in our hospital from 1999 to 2002 [18]. The spectrum of causative organisms and related antibiotic susceptibility were similar in both studies. This information is very helpful in choosing adequate empirical antimicrobial agents for BSI, especially in patients with high risk of mortality.

Despite beyond the scope of current study, several studies have indicated that novel anti-myeloma agents may reduce early mortality. Tan et al. demonstrated that the frontline use of bortezomib compared to its sequential may avert early mortality (death within 2 years of diagnosis), especially among patients with high-risk MM [19]. Kumar et al. found that early mortality (death within 1 year of diagnosis) was significantly lower among the patients who had received one of the newer drugs as part of their initial therapy (8% vs. 19%; *p* < 0.001) [20]. They also identified age > 70, serum albumin < 3.5 gm/dL, and serum beta_2_ microglobulin > 6.5 mg/dL as factors independently predicting early mortality [20]. These three parameters are similar to advanced ISS stage and poor ECOG performance status proposed in our study.

There are several limitations of our study, first is the retrospective design of the study which may have patient and treatment selection biases. Secondly, the study period is between November 2002 and December 2008. The fact that very few patients receiving newer induction agents (such as proteasome inhibitors and/or immunomodulating drugs limits our general application to myeloma patients who are now receiving these newer agents as induction therapy. Due to the individualized decisions on the induction regimens, it is difficult for us to analyze the impact of newer agents on bloodstream infections. Moreover, in countries with limited health resources, the traditional therapies remain the backbone of combination therapy for multiple myeloma, and novel agents may not be permitted as first-line therapy. Our results need further validation in the era of novel agents. In our study, early mortality was defined arbitrarily as death within 100 days to approximately estimate the impact of bloodstream infections on outcome. Therefore, individual specific cause of deaths, as well as competing death events could not be assessed in the survival analysis. In addition, the impact of BSI on outcome could not be assessed on patients developing BSI within 90 days but dying after 100 days, although these patients were also likely died of infections.

## Conclusions

In conclusion, we stress that BSI in newly diagnosed MM patients is a frequently observed clinical condition that requires prompt recognition and adequate antimicrobial treatment, outside of anti-myeloma therapies, to reduce the risk of early mortality. Patients with more advanced ISS stage and poor ECOG performance status should be classified as a high risk group. Epidemiological data of common pathogens with antibiotic susceptibility is useful in improving clinical decision-making. More prospective studies are required to confirm the impact of the frontline use of novel agents in early mortality of MM and the accuracy of the developed scoring system to predict the risk of BSI as well as the role of antimicrobial prophylaxis in newly diagnosed patients.

## Additional file

Additional file 1. Antibiotics susceptibility of blood stream infections. (DOCX 16 kb)

## Abbreviations

ALC: Absolute lymphocyte count
BSI: Blood stream infection
Ca: Calcium
CI: Confidence interval
CoNS: Coagulase-negative staphylococcus
Cr: Creatinine
CVC: Central venous catheter
ECOG PS: Eastern Cooperative Oncology Group Performance Status
Hb: Hemoglobin
ICT: Induction chemotherapy
Ig: Immunoglobulin
IgA: Immunoglobulin A
IgG: Immunoglobulin G
ISS stage: International Staging System
MP: Melphalan/prednisolone
MPT: Melphalan/prednisolone/thalidomide
PICC: Peripherally inserted central catheter
R: Resistant
VAD: Vincristine/doxorubicin/dexamethasone
