# Supplementary material for: Risk factors and characteristics of blood stream infections in patients with newly diagnosed multiple myeloma
Source: BMC Infect Dis. 2017 Jan 6;17:33. doi: 10.1186/s12879-016-2155-1 (PMC5217598; doi:10.1186/s12879-016-2155-1)
Supplement: Additional file 1. — Antibiotics susceptibility of blood stream infections. (DOCX 16 kb) [file 12879_2016_2155_MOESM1_ESM.docx]

# Antibiotics susceptibility of blood stream infections

| **Gram positive** | **Total**  **Isolates, n** | **Ampicillin–R n (%)** | **Cefazolin-R**  **n (%)** | **Vancomycin-R**  **n (%)** |  |  |  |  |
| --- | --- | --- | --- | --- | --- | --- | --- | --- |
| Gram positive pathogens | **11 (11/27)** | 7 (70) | 6 (60) | 0 (0) |  |  |  |  |
| *CoNS*  *Staphylococcus aureus*  *Streptococcus pneumoniae* | 6  2  2 | 5 (100)  2 (100)  0 (0) | 4 (80)  2 (100)  0 (0) | 0 (0)  0 (0)  0 (0) |  |  |  |  |
| *Lactobacillus species* | 1 | 0 (0) | 0 (0) | 0 (0) |  |  |  |  |
| **Gram Negative** |  | **Ampicillin/ sulbactam−R, n (%)** | **Cefuroxime−R**  **n (%)** | **Ceftazidime−R**  **n (%)** | **Cefepime−R**  **n (%)** | **Amikacin-R**  **n (%)** | **Ciprofloxacin-R**  **n (%)** | **Imipenem-R**  **n (%)** |
| **Gram Negative Pathogens**  *Escherichia coli* | **15 (15/27)**  5 | 5 (33)  1 (20) | 4 (27)  0 (0) | 2 (13)  0 (0) | 1 (7)  0 (0) | 1 (7)  0 (0) | 4 (27)  1 (20) | 0 (0)  0 (0) |
| *Klebsiella pneumoniae*  *Enterobacter* species | 2  2 | 2 (67)  1 (50) | 2 (67)  0 (0) | 2 (67)  0 (0) | 1 (33)  0 (0) | 1 (33)  0 (0) | 2 (66)  0 (0) | 0 (0)  0 (0) |
| *Pseudomonas aeruginosa* | 2 | 1 (50) | 2 (100) | 0 (0) | 0 (0) | 0 (0) | 1 (50) | 0 (0) |
| *Proteus mirabilis*  *Salmonella enteritidis group D*  *Serratia marcescens* | 1  1  1 | 0 (0)  0 (0)  0 (0) | 0 (0)  0 (0)  0 (0) | 0 (0)  0 (0)  0 (0) | 0 (0)  0 (0)  0 (0) | 0 (0)  0 (0)  0 (0) | 0 (0)  0 (0)  0 (0) | 0 (0)  0 (0)  0 (0) |
| **Fungus** |  | **Fluconazole-R** | **Itraconazole-R** |  |  |  |  |  |
| *Candida albicans*  *Trichosporon asahii* | 1  1 | NA  0 (0) | NA  0 (0) |  |  |  |  |  |
| Abbreviations: *CoNS* Coagulase-negative staphylococcus, *R* Resistant | | | | | | | | |
